# Photoactivatable Caged Prodrugs of VEGFR-2 Kinase Inhibitors

**DOI:** 10.3390/molecules21050570

**Published:** 2016-04-29

**Authors:** Boris Pinchuk, Rebecca Horbert, Alexander Döbber, Lydia Kuhl, Christian Peifer

**Affiliations:** Institute of Pharmacy, University of Kiel, Gutenbergstr. 76, D-24118 Kiel, Germany; bpinchuk@pharmazie.uni-kiel.de (B.P.); rhorbert@pharmazie.uni-kiel.de (R.H.); adoebber@pharmazie.uni-kiel.de (A.D.); lkuhl@pharmazie.uni-kiel.de (L.K.)

**Keywords:** photoactivatable prodrugs, caging, receptor tyrosine kinase, kinase inhibitors, VEGFR-2, 3,4-diarylmaleimides, photoremovable protecting group (PPG)

## Abstract

In this study, we report on the design, synthesis, photokinetic properties and *in vitro* evaluation of photoactivatable caged prodrugs for the receptor tyrosine kinase VEGFR-2. Highly potent VEGFR-2 inhibitors **1** and **3** were caged by introduction of a photoremovable protecting group (PPG) to yield the caged prodrugs **4** and **5**. As expected, enzymatic and cellular proliferation assays showed dramatically diminished efficacy of caged prodrugs *in vitro*. Upon ultraviolet (UV) irradiation of the prodrugs original inhibitory activity was completely restored and even distinctly reinforced, as was the case for the prodrug **4**. The presented results are a further evidence for caging technique being an interesting approach in the protein kinase field. It could enable spatial and temporal control for the inhibition of VEGFR-2. The described photoactivatable prodrugs might be highly useful as biological probes for studying the VEGFR-2 signal transduction.

## 1. Introduction

Among protein kinases (PK), receptor tyrosine kinases such as vascular endothelial growth factor receptor (VEGFR) are significant drug targets for the development of clinically effective small molecule kinase inhibitors (smKI) [1,2,3]. VEGFR-2 plays an important role in the physiological regulation of angiogenesis [4]. This receptor tyrosine kinase is one of the key mediators of pathophysiological formation of blood vessels associated with tumor growth and intraocular neovascular diseases [5,6,7,8]. Besides therapeutic antibodies [9], small molecule tyrosine kinase inhibitors (TKIs) targeting VEGFR have been developed for the treatment of various neoplastic diseases [10,11,12,13,14,15]. Seven VEGFR inhibitors have been approved until 2016 [16]. However, TKI are often promiscuous, showing significant affinity for related tyrosine kinases such as fibroblast growth factor receptor (FGF-R), epidermal growth factor receptor (EGF-R), platelet derived growth factor receptors (PDGFRα/β), c-kit, Src, and for further PK [17,18]. The lack of specificity and off-target effects often limit the therapeutic value of such inhibitors. Besides cancer, VEGFR mediated angiogenesis is also critical in inflammation, wound healing, cardiovascular diseases, psoriasis, rheumatoid arthritis, and macular degeneration [7,19,20,21,22,23]. The impressive number of diseases illustrates the enormous potential for therapeutic agents and for biological probes in VEGFR research or related biological chemistry applications.

**Scheme 1:**
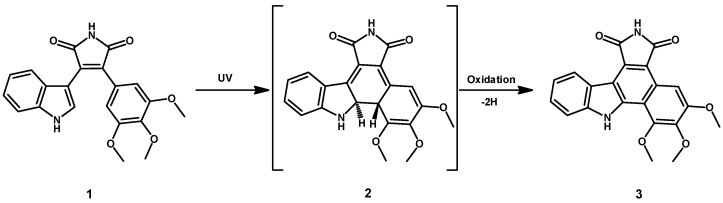
Ultraviolet (UV)-induced 1,6-π-electrocyclization of the 3,4-diarylmaleimide **1** followed by oxidation to the carbazole derivate **3** [25]. The intermediate **2** could not be isolated.

In 2006 a novel class of potent VEGFR inhibitors, namely 3,4-diarylmaleimides, was reported by Peifer *et al.* [24,25]. Within this series compound **1** showed the highest potency with an IC_50_ towards VEGFR-2 of 2.5 nM [24]. The 1,6-π-electrocyclization of **1** followed by subsequent oxidation to carbazole **3** caused by UV-irradiation was also described (Scheme 1) [25].

Both compounds, **1** and **3**, were found to be potent and specific VEGFR-2 inhibitors with strong anti-angiogenetic activity (VEGFR-2, IC_50_ = 2.5 nM and 62 nM for **1** and **3**, respectively) [25]. In light of the immense significance of VEGFR-2 inhibitors we aimed to develop relevant photoactivatable caged VEGFR-2 prodrugs. An approach using photoremovable protecting groups (PPG) provides spatial and temporal control over the release of a bioactive molecule by irradiation with UV light [26,27,28]. The bioactive inhibitor can be generated at a defined time point in an irradiated area of interest. Caged VEGFR-2 prodrugs could serve as novel experimental tools, e.g., for kinetic or mechanistic studies. Moreover, caged inhibitors should minimize systemic side effects. This might enable higher dosage of inactive prodrugs. Consequently, controllable irradiation should increase the concentration of the active drug in a cancer-afflicted tissue sharply.

**Figure 1:**
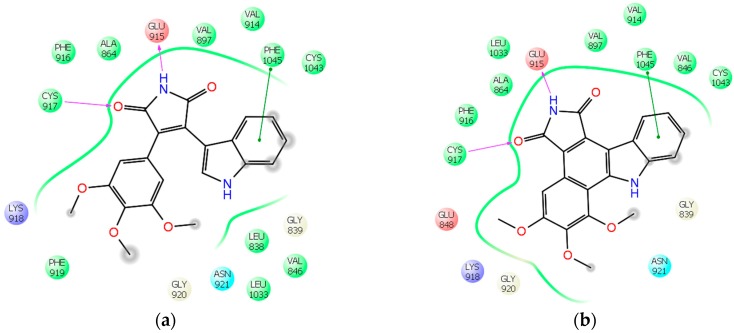
Modeled ligand interaction diagrams of VEGFR-2 inhibitors **1** and **3** in the ATP binding pocket of VEGFR-2 (pdb code 3CJF). Key ligand protein interactions are shown including H-bonds of the maleimide moiety towards Glu915 and Cys917 in the hinge region. (**a**) Binding mode of **1**; (**b**) Binding mode of **3**.

A caged prodrug is typically designed by blocking a crucial pharmacophore moiety of the inhibitor using a PPG. Regarding smKI, this is most effectively done by blocking the hinge binder as this motif is basically used by all type I/II inhibitors [29]. Preventing a smKI from binding to the central hinge region not only renders the compound biologically inactive against the PK of interest but most likely against all other PK as well [30]. The modeled binding modes of **1** and **3** in the ATP binding site of VEGFR-2 were previously described [24]. Key interactions between the ligand and the protein are the H-bonds of the maleimide moiety towards the hinge region as shown in Figure 1.

**Figure 2:**
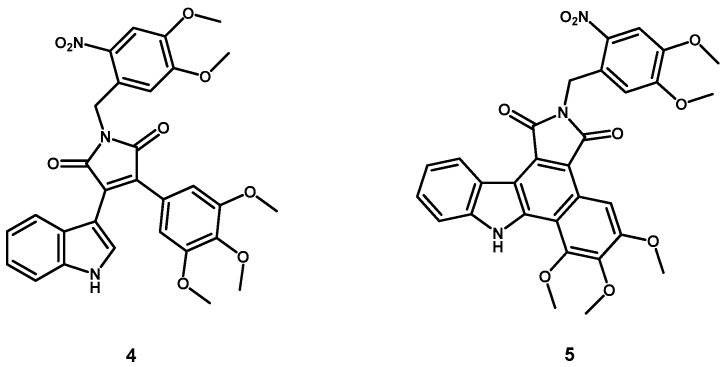
Designed caged compounds **4** and **5** with the 4,5-dimethoxy-2-nitrobenzyl (DMNB) protecting group attached to the maleimide resp. carbazole in order to prevent hinge binding.

Among PPGs, *o*-nitrobenzylic derivatives (*o*-NB) have been successfully used in various biological applications [26,28,31,32,33]. The 4,5-dimethoxy-2-nitrobenzyl (DMNB) protecting group is a variation of *o*-NB that can be cleaved by irradiation at 365 nm [34]. This wavelength is less energetic compared to 254 nm used for the cleavage of *o*-NB, so extensive cell damage can be avoided [35]. There are some recent examples for the use of DMNB including photoactivatable protein antigens for controlling the antibody-antigen interactions [36], a caged transmitter for studying neuron–glia interactions [37], and caged abscisic acid used to promote photo-induced protein dimerization [38]. We applied the DMNB caged concept to the key maleimide hinge binding motif of VEGFR-2 inhibitors **1** and **3** by designing compounds **4** and **5**, respectively, in which the relevant hinge binding NH moiety is blocked (Figure 2).

Herein, we report on the design, synthesis, photochemical characterization and biological evaluation of the compounds **4** and **5**. These compounds are photoactivatable prodrugs of the previously described VEGFR-2 inhibitors **1** and **3**, respectively. First, molecular modeling studies predicted that blocking the maleimide moiety of the active compounds should diminish the inhibitory efficacy. Having the caged prodrugs synthesized, we next investigated the photoinduced cleavage of PPG and the release of the native inhibitors. The loss-off-function by photoprotection was proven *in vitro* both in enzymatic and in cellular proliferation assays. Finally, reconstitution of the inhibitory activity by UV irradiation has been demonstrated in cellular assays. The here presented photoactivatable prodrugs of VEGFR-2 inhibitors could be used as a novel pharmacological approach in VEGF-signaling research.

## 2. Results

### 2.1. Molecular Modeling

Molecular docking of the active compounds **1** and **3** into the ATP binding site of VEGFR-2 (pdb code 3CJF) revealed the maleimide moiety as the key pharmacophore group for the inhibitors interaction towards the hinge region of the target protein (Figure 1). To prove our prodrug concept we additionally docked caged **4** and **5** into the same pocket. In accordance with our hypothesis, the latter docking experiment did not result in plausible binding modes of the caged compounds in the active site (not shown). The DMNB protecting group prevented key H-bond-interactions to the hinge region. Moreover, the caged compounds did not fit into the binding pocket due to sterical clashes. Motivated by modeling results we synthesized **4** and **5** and subsequently characterized these compounds for their photochemical properties to determine parameters for decaging and potential usability for biological evaluation.

### 2.2. Synthesis

**Scheme 2:**
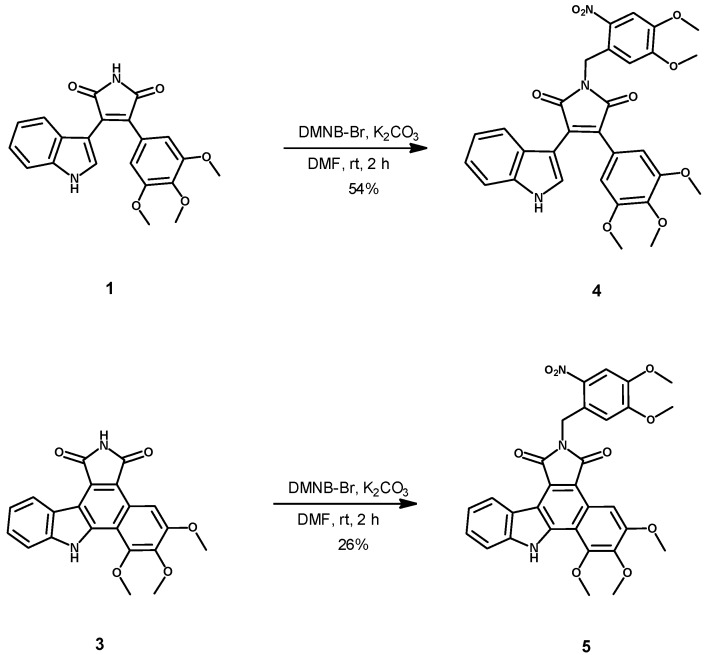
Synthesis of **4** and **5** [25,39].

Compounds **1** and **3** were synthesized by literature procedures [25,39]. The synthesis of the caged compounds **4** and **5** from **1** and **3**, respectively, was found to proceed straightforward in terms of a base catalyzed S_N_ reaction by deprotonation of the acidic maleimide moiety, and using DMNB-Br as a reactant (Scheme 2).

### 2.3. Photochemical Characterization

**Figure 3:**
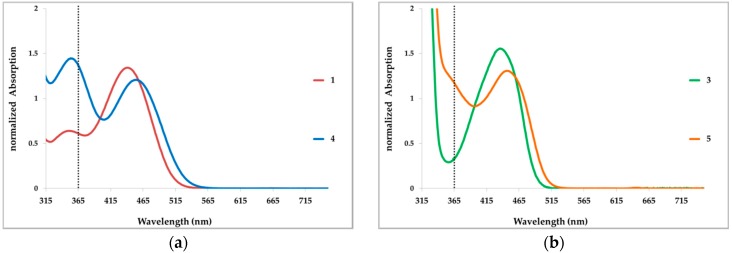
Normalized UV/Vis absorption spectra of compounds in DMSO. (**a**) UV/Vis absorption spectra of maleimide **1** (red line) and its caged prodrug **4** (blue line); (**b**) UV/Vis absorption spectra of carbazole **3** (green line) and its caged analogue **5** (orange line). The black dotted line in both diagrams flags 365 nm as the wavelength used for irradiation of caged compounds.

Having both active and caged compounds, we investigated their photochemical characteristics. First, we recorded the UV/Vis absorption spectra both for maleimide and carbazole derivatives before and after insertion of the DMNB group, to find an appropriate wavelength for PPG cleavage. The normalized spectra are shown in Figure 3. The raw spectra can be found in the Supplementary Materials (Figure S1).

As shown in Figure 3, introduction of the DMNB PPG leads to increased light absorption around 365 nm (black dotted line). This applies for maleimides (Figure 3a) and carbazoles (Figure 3b). The same wavelength was previously described for the cleavage of the inserted DMNB group [27]. Wavelengths shorter than 300 nm are highly energetic and can easily damage biological tissues. 365 nm can therefore be considered as the optimal wavelength for deprotection. Furthermore, the inserted PPG in **4** and **5** causes a weak bathochromic spectral shift of these compounds. This effect can be explained by an increased electron density due to substitution of the hydrogen at the imide nitrogen by the DMNB protecting group.

**Figure 4:**
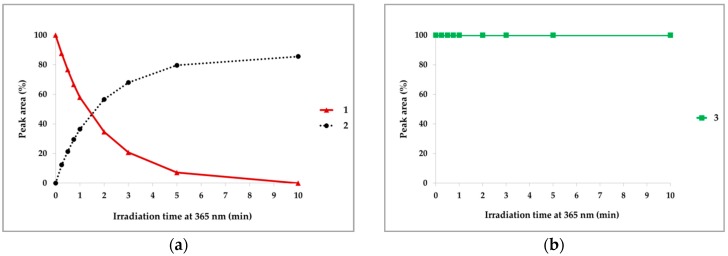
UV stability of the parent VEGFR-2 inhibitors. The compounds were irradiated at 365 nm with 5.4 W for up to 10 min and analyzed time dependently by HPLC. The detection wavelength for the HPLC analysis was 300 nm. The area under the HPLC-peaks is plotted against the irradiation time. (**a**) Irradiation of 1 mM solution of **1** in DMSO (red line). By progressing irradiation, **1** is converted into the intermediate product **2** (dotted black line); (**b**) Irradiation of 1 mM solution of **3** in DMSO (green line). **3** is stable under the described UV irradiation.

In general, one of the preconditions for successful caging approaches is the stability of the parent compounds when irradiated at the wavelength used for PPG cleavage. This stability is crucial, otherwise the actual inhibitor molecule would be degraded immediately after its release from the caged compound. For this reason, we next examined the stability of **1** and **3** towards irradiation at 365 nm. The light-emitting diode (LED) reactor with a wavelength of 365 nm (5.4 W; for technical information, see Supplementary Materials Figures S2 and S3) was used to irradiate 1 mM solutions of **1** and **3** in DMSO (Figure 4). HPLC and LC-MS analysis were used for content determination.

Irradiation of **1** at 365 nm induced the 1,6-π-electrocyclization of the 3,4-diarylmaleimide **1** to the intermediate **2** as analyzed by LC-MS (also see Scheme 1). Under the used irradiation conditions the subsequent oxidation to carbazole **3** did not occur (Figure 4a). Resulting from its instability at 365 nm, the maleimide **1** is not optimally suitable for the photocaging concept. In contrast, the carbazole **3** is quite stable under irradiation by 365 nm (Supplementary Figure S1b). Based on this data, the carbazole derivative **3** is more eligible for the caging concept than the 3,4-diarylmaleimide **1**.

**Figure 5:**
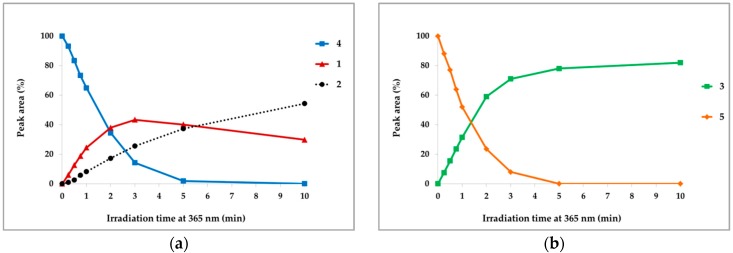
Release of parent VEGFR-2 inhibitors by irradiating their caged prodrugs. The DMNB-protected derivatives were irradiated at 365 nm with 5.4 W for up to 10 min. The uncaging was examined by HPLC and LC-MS analysis. The detection wavelength for the HPLC analysis was 300 nm. The amount of released parent compounds and their caged prodrugs are plotted as areas under HPLC-peaks against irradiation time. (**a**) Irradiation of 1 mM solution of **4** in DMSO (blue line) yields initially the maleimide **1** (red line). Continuing UV exposure leads to 1,6-π-electrocyclization of **1** to **2** (dotted black line); (**b**) Irradiation of 1 mM solution of **5** in DMSO (orange line) results in the clean release of carbazole **3** (green line).

A further prerequisite for an application of caged prodrugs is a rapid and quantitative release of active parent compounds by irradiation. To examine this, we investigated the uncaging of **4** and **5** induced by irradiation at 365 nm with 5.4 W (Figure 5). Exposure to UV light caused the cleavage of the DMNB PPG from caged compound **4**. Initially released maleimide **1** is converted to the intermediate **2** by proceeding irradiation, which is in line to the previously described instability of **1**, (compare Figure 4a). As expected, carbazole **3** was rapidly released from the caged derivative **5** by irradiating at 365 nm. Due to UV stability of **3** no other by-products could be detected.

Due to the results of photochemical characterization, it can be summarized that the caged carbazole **5** was a suitable candidate for our VEGFR-2 inhibitor caging concept. The straightforward release of parent compound **3** by short UV irradiation and the UV stability of uncaged molecule made the carbazoles interesting for biological evaluation *in vitro*. Although the maleimide **1** does not possess appropriate UV stability, we tested the caged **4** *in vitro* to determine its cellular efficacy too.

### 2.4. Enzyme Assays

As predicted by molecular modeling, insertion of the protecting group DMNB should diminish the inhibitory activity both of the maleimide **1** and the carbazole **3**. To prove this hypothesis, active compounds **1** and **3** and caged prodrugs **4** and **5** were tested in a radiometric kinase assay [40]. The IC_50_ values towards VEGFR-2 were determined (Table 1).

The IC_50_ value of **1** is in low nanomolar range (IC_50_ = 0.005 µM) and is comparable with the previously determined value [24]. The carbazole derivative **3** is an effective VEGFR-2 inhibitor too, although it is less active than **1** (IC_50_ = 0.152 µM). Moreover, the measured IC_50_ values confirm that the caging significantly reduces the compounds activity. The caged compounds have at least a two magnitudes lower efficacy than the active inhibitors. The determined IC_50_ values are 4.59 µM and 44.8 µM for **4** and **5**, respectively. The minimal residual activity of the caged compounds can be explained by minor impurities of active inhibitors in the samples. To sum up, we verified that the insertion of the DMNB PPG significantly decreases the inhibitory efficacy both of maleimides and of carbazoles.

**Figure 6:**
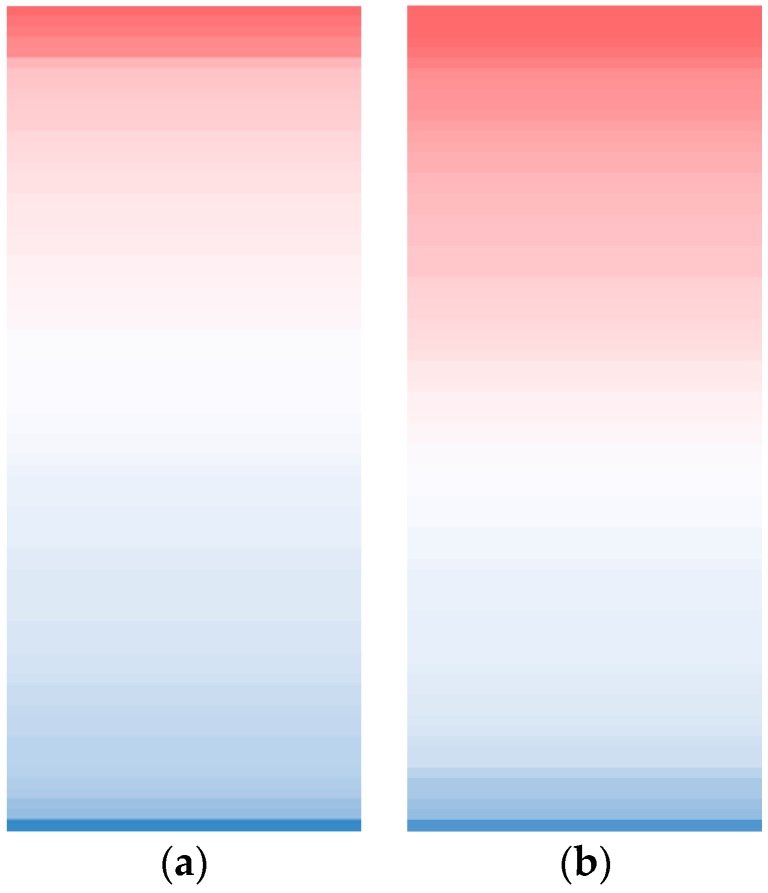
Selectivity profile of active compounds in a panel of 79 kinases. The inhibitory effect was examined at inhibitor’s concentration of 0.1 µM by determination of residual kinase activity. The data are presented as a heatmap. The columns have been individually ranked from low (red) to high (blue) residual activity values. The raw data are shown in the Supplementary Table S1. (**a**) Selectivity profile of **1**; (**b**) Selectivity profile of **3** (MRC, Dundee, Scotland).

To compare the active inhibitors **1** and **3**, a selectivity profile over 79 kinases was recorded [41,42]. The residual kinase activities are presented as a heatmap (Figure 6, for raw data see Supplementary Table S1). The selectivity profile shows that the diarylmaleimide **1** is more selective than its carbazole derivative **3**.

### 2.5. Cellular Assays

Due to the findings described above, we supposed that the caged molecules **4** and **5** would be considerably less active in cellular assays as well compared to the parent compounds **1** and **3**. To prove this hypothesis, we examined their antiproliferative activity *in vitro*. Growth assays on VEGFR-2 dependent PC-3 cells [43,44,45] were performed.

**Figure 7:**
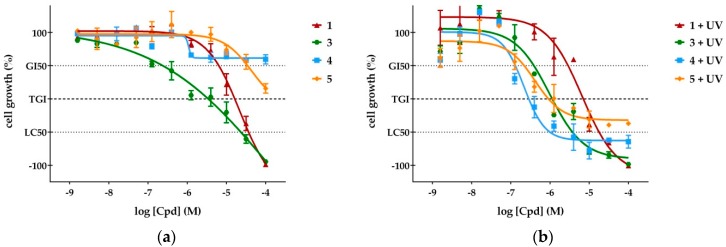
Antiproliferative activity of tested compounds on VEGFR-2 dependent PC-3 cells. (**a**) Dose-response curves of active inhibitors (**1** and **3**) and their corresponding caged compounds (**4** and **5**) were determined without UV irradiation. Cell growth was measured 48 h after incubation with the compounds. The caged derivatives do not exhibit cytotoxic activity: the TGI mark is not reached even at high concentrations; (**b**) Cells were incubated for 1 h with the compounds and then irradiated at 365 nm (1.1 kW/m²) for 5 min. Cell growth was determined 48 h after incubation with the compounds. After irradiation, the caged derivatives show similar (**5**) or even left shifted (**4**) dose-response curves as that of active compounds. GI50 = 50% growth inhibition; TGI = total growth inhibition; LC_50_ = 50% lethal concentration; *n* = 4. Error bars represent standard deviation. The effect of UV irradiation on the PC-3 cells is shown in Supplementary Figure S4.

Dose-response curves for non-irradiated compounds are shown in Figure 7a. The corresponding GI_50_ values (compounds concentration at which the cellular growth is inhibited by 50%) are shown in Table 2. Compounds **1** and **3** exhibit potent antiproliferative efficacy at concentrations in the low µM range. The GI50 value for the maleimide **1** was measured as 6.4 µM. In contrast to **1**, its caged derivative **4** exhibits very marginal antiproliferative activity, even at high concentrations the GI_50_ for **4** was not reached. The carbazole **3** (GI_50_ = 0.2 µM) is more potent in this assay than the maleimide **1**. This increased cytostatic activity could be caused by promiscuity of **3** that inhibits more kinases than **1** and therefore induces higher stress for the exposed cells. This unexpected cellular response to **3** should be examined in greater depth. Once again, the caged derivative **5** was significantly less active (GI_50_ = 34.6 µM) than its parent compound **3**. The low cytostatic activity of the caged compounds at high concentrations might be explained by minor impurities of non-caged parent compounds in the samples.

Dose-response curves for non-irradiated compounds are displayed in After proving the diminished cellular activity of caged compounds, we next explored if their antiproliferative efficacy could be restored by UV irradiation. The cellular growth assays described above were repeated and the compound treated PC-3 cells were exposed to UV light at 365 m (5 min, 1.1 kW/m^2^). The measured dose-response curves are shown in Figure 7b. The corresponding GI_50_ values are listed in Table 2. Previously, control experiments verified that the PC-3 cells tolerate the applied dosage of UV irradiation sufficiently well; the cellular growth is reduced only to 90% by used irradiation (Supplementary Figure S4). The determined dose-response curves demonstrate that 5 min exposure to UV light completely restores the inhibitory activity of the caged compounds. After irradiation, the caged derivatives exhibit comparable or even stronger antiproliferative efficacy compared to that of their not caged parent compounds. The detected GI_50_ value of the irradiated **1** (2.9 µM) is slightly lower than of non-irradiated **1** (6.4 µM). However, a significantly increased inhibitory effect could be detected for the irradiated caged **4** (GI_50_ value was 0.1 µM). Interestingly, the caged molecule **4** is about 10-fold more active after UV treatment than its parent maleimide **1**. One of the possible reasons for this increased activity might be a biological effect of the released protecting group. To investigate the effect of the cleaved PPG moiety, we used two model compounds: Boc-protected l-alanine (Boc-Ala) and its DMNB photo protected derivative (DMNB-Boc-Ala), as we considered Boc-Ala to be nontoxic. First, the effect of UV unexposed compounds on proliferation of the PC-3 cells was explored. Both protected amino acid derivatives did not show any antiproliferative effects even at high concentrations (Supplementary Figure S5c). The same experiment was repeated with UV irradiation (365 nm, 5 min, 1.1 kW/m^2^). UV exposed Boc-Ala does not have any effect on cellular growth. In contrast, DMNB-Boc-Ala exhibits distinct antiproliferative activity after irradiation at concentrations above 10 µM (GI_50_ = 50.3 µM) (Supplementary Figure S5d). Therefore, it could be assumed that the antiproliferative activity in this assay is caused by the cleaved DMNB. However, the concentration at which the PPG shows toxicity (above 10 µM) is approximately 500-fold higher than the efficacious concentration of the irradiated **4**. Thus, it is not only the cleaved PPG that is responsible for dramatically increased activity of UV-exposed **4**. Presumably, the following parameters contribute to the biological efficacy of irradiated **4**: The released active inhibitor **1**, the formation of the intermediate **2** (Figure 5a), the cleaved PPG and, finally, the UV irradiation itself cause the described rise of activity in synergistic manner. After irradiation, the carbazole 3 displayed a similar efficacy to that before UV exposure: GI_50_ value was 0.38 µM. The activity of its caged derivative **5** was completely restored by irradiation (GI_50_ = 0.22 µM).

Summing up, we demonstrated that the insertion of the DMNB PPG effectively diminishes the activity of the inhibitors **1** and **3**. The antiproliferative efficacy can be restored easily or even reinforced by UV irradiation of the corresponding caged compounds **4** and **5**.

**Figure 8:**
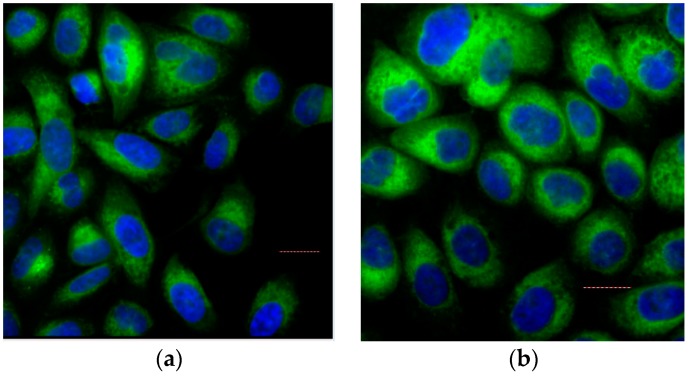
Microscopic images of stained PC-3 cells. The investigated compounds **3** and **5** are shown in green. Both compounds were applied at 10 µM concentrations. The cell nuclei were counterstained with 1 µg/mL DAPI and are marked blue. The dashed line represents 20.3 µm. (**a**) Staining with carbazole **3**; (**b**) Staining with caged compound **5**.

Next, we investigated the cellular bioavailability of the caged prodrugs. For this purpose, we used the autofluorescence of the carbazole **3** and its caged derivative **5**. The emission spectra of both compounds are shown in the Supplementary Figure S6. The fluorescence microscopy was used to explore the uptake of the compounds into the cells. Live PC-3 cells were stained with 10 µM solutions of **3** or **5**, and then fixed with formalin. Subsequently, the cell nuclei were counterstained with 1 µg/mL DAPI. As demonstrated by fluorescence microscopy (Figure 8) both carbazoles **3** and **5** are taken up into the cells, but not within the cell nuclei. The insertion of the DMNB PPG does not alter the cellular uptake of the carbazoles (Figure 8b). Due to this data, it can be concluded that the DMNB PPG does not decrease the cell permeability of the inhibitors.

## 3. Discussion

There are examples of photoactivatable kinase inhibitors described in the literature [31,35,46]. The interest in this research field is motivated by exciting options offered by the caging concept in basic research as well as in therapeutic applications. Targeted irradiation enables release of active inhibitors at higher concentrations in a precisely controlled way. Temporally and locally verifiable activation is a powerful pharmacological tool to study the biological effects of inhibiting kinase signaling. Moreover, this caging approach could spare undesirable side effects associated with kinase inhibitor therapy.

Our research group has already evaluated caged derivatives of the approved kinase inhibitors vemurafenib [30] and imatinib [47] *in vitro*. To the best of our knowledge, there are no caged VEGFR-2 inhibitors described so far. Therefore, it was our motivation to verify the caging concept on this therapeutically important kinase. In this article, we report on the design, synthesis, photochemical and *in vitro* characterization of previously published VEGFR-2 inhibitors **1** and **3** [24,25]. By usage of DMNB as the PPG, we synthesized caged derivatives **4** and **5**. These novel photoactivatable prodrugs of VEGFR-2 inhibitors can be used in a variety of experiments investigating the VEGF-signaling. Irradiation of the caged maleimide **4** did not result in a clean release of the parent inhibitor **1** but yielded a mixture of **1** and **2**. The intermediate **2** could not be isolated but it could have some unexplored biological effects *in situ* due to its oxidation to **3**. Another interesting result was the increased antiproliferative activity of **4** after irradiation. The cytotoxic activity of **4** after UV exposure was 10-fold higher in cellular assays compared to its parent compound **1**. This observation cannot be explained separately whether by the formation of intermediate **2** nor by the cleaved PPG. The anticipated formation of **2** alone is unlikely to cause this gain of activity, because it was not observed during irradiation of **1** under the same experimental conditions. By usage of model compounds it could be demonstrated that the cleaved DMNB group exhibit antiproliferative activity only in concentrations above 10 µM. Presumably, there are some synergistic effects of the released inhibitor, the generated intermediate, the cleaved PPG and the UV irradiation that cause the increased efficacy of irradiated **4**. On the one hand, this increased activity could promote the usage of **4** as a research tool for VEGF-signaling. On the other hand, the distinct gain of antiproliferative efficacy towards cancer cells can be very interesting for possible medicinal applications. In contrast to maleimides, the photoinduced release of active carbazole **3** from its caged derivative **5** succeeded clearly, fast, and without formation of observable byproducts. The insertion of the DMNB group did not prevent the cellular uptake of the carbazole. Therefore, caged carbazole **5** provides a photoactivatable VEGFR-2 inhibitor that can be used as a valuable tool for studying VEGF-signaling.

The implementation of DMNB caged kinase inhibitors in therapeutically relevant approaches might be restricted due to necessity of UV light for the release of active compounds. Although UVA irradiation (wavelengths above 320 nm) causes less direct DNA damage than UVB or UVC exposure [48], high dosage of UVA is toxic and can lead to oxidative stress, photoaging, and immunosuppression [48,49]. Another restriction for a medical application of caged prodrugs is the poor penetration of UV light into biological tissues. Only depths of about 100 µm can be reached [50]. There are several possible solutions to overcome the poor penetration: optical fibers or endoscopic probes can transmit the required light to the site of action. Moreover, nearby areas could be irradiated during surgery.

One option to dispense with UV light is the usage of protecting groups that are cleavable by the visible light irradiation. Some current examples of these groups are BODIPY derived PPGs and thiocoumarins. BODIPY-based protecting groups can be removed with green wavelengths above 500 nm [51]. Irradiation with cyan light can cleave thionated coumarins [52]. The application of visible light for uncaging would enable nontoxic release of active compounds in deeper tissue layers. The best permeation through biological tissues can be achieved within the biological optical window by wavelengths around 800 nm [53]. Hence, two-photon excitation is an interesting approach, even though it requires special equipment like fs-pulsed lasers [54]. It remains to be seen if these novel developments would allow a medical application of the caged compounds.

## 4. Materials and Methods

### 4.1. Molecular Modeling

Molecular modeling was performed on a DELL 8 core system. For visualization Maestro, version 9.7, Schrödinger, LLC, (New York, NY, USA, 2014) was used. Protein crystal structures were prepared prior to docking by the Protein Preparation Wizard [55] utilizing the following programs: Epik, version 2.7, 2014 [56]; Prime, version 2.4, 2014 [57]. Thus, the X-ray crystal structure refinement process included addition of hydrogen atoms, optimization of hydrogen bonds, and removal of atomic clashes. Default settings were used. Missing side chains and loops were filled in with Prime. Furthermore, selenomethionines were converted to methionines and water molecules were deleted.

Additionally, ligands were prepared in order to create energetically minimized 3D geometries and assign proper bond orders (MacroModel, version 10.3, 2014 [58]). Accessible tautomer and ionization states were calculated prior to screening (LigPrep, version 2.9, 2014 [59]). To generate bioactive conformers a conformational search method was used (ConfGen, version 2.7, 2014 [60]). Receptor grid generation was performed by Glide, version 6.2, 2014 [61]. For ligand docking and screening the Glide SP workflow was used. Energetically minimized ligand conformations were docked into the active site of the protein; possible binding poses were determined and subsequently ranked based on their calculated binding affinities.

### 4.2. Chemistry

All reagents and solvents were obtained from commercial sources and used as received (THF was used after distillation over K/benzophenone). Reagents were purchased from abcr GmbH (Karlsruhe, Germany), Fisher Scientific GmbH/Acros (Schwerte, Germany), Sigma-Aldrich Chemie (Hamburg, Germany) or VWR International GmbH (Hannover, Germany).

Where appropriate, column chromatography was performed for crude precursors with Merck (Darmstadt, Germany) silica gel 60 (0.063–0.200 mm) or Acros Organics silica gel (0.060–0.200 mm; pore diameter *ca.* 60 nm). Column chromatography was performed on a LaFlash system (VWR) using silica gel columns (PF-30SIHP, 30 μm, 40 g, puriFlash) or RP18 columns (PF-15C18HP, 15 μm, 55 g, puriFlash). The crude product was loaded on Merck silica gel 60 (15–40 μm). The progress of reactions was monitored by thin-layer chromatography (TLC) utilizing silica gel polyester sheets (SIL G/UV254, 0.2 mm, Polygram^®^, Macherey-Nagel GmbH (Düren, Germany).

High-performance liquid chromatography (HPLC) analyses were performed on a 1050 Series system (Hewlett Packard, Ratingen, Germany). As column an Agilent ZORBAX^®^ Eclipse XDB-C8, 5 μm (4.6 mm × 150 mm) was used. Injection volume of the compound solutions was 20 μL. As mobile phase (flow rate 1.5 mL/min) served a gradient of KH_2_PO_4_ buffer (10 mM, pH 2.3) and methanol over 14 min. The detection wavelength was adapted to the according UV/vis absorption spectra. All key compounds submitted to biological assays were proven by this method to show ≥98% purity.

Melting points were determined on a SMP3 apparatus (Stuart Scientific, Staffordshire, UK) and are uncorrected. ^1^H- (300 MHz) and ^13^C- (75 MHz) NMR were recorded on an Avance III 300 spectrometer (Bruker, Rheinstetten, Germany) at 300 K with a multinuclear probe head using the manufacturer´s pulse programs. The data are reported as follows: chemical shifts in ppm from Me_4_Si (TMS) as external standard, multiplicity and coupling constant (Hz). NMR spectra were obtained on a ^1^H (300 MHz) and ^13^C spectra (75 MHz) were referenced either to TMS or to internal DMSO-*d*_5_ (^1^H-NMR δ 2.50) and internal DMSO-*d*_6_ (^13^C-NMR δ 39.5) or internal CHCl_3_ (^1^H-NMR δ 7.26) and internal CDCl_3_ (^13^C-NMR δ 77.0). All coupling constants (*J* values) are quoted in Hz. The following NMR abbreviations are used: b (broad), s (singlet), d (doublet), t (triplet), m (unresolved multiplet). The labeling scheme of structures to correlate NMR signals is included in the data.

LC-MS samples were chromatographically separated utilizing a 1100 HPLC system (Agilent, Waldbronn, Germany) consisting of a thermostated autosampler, diode array detection, and an Agilent ZORBAX^®^ Eclipse XDB-C8, 5 μm (4.6 mm × 150 mm). Elution was achieved with a solvent gradient system of water and acetonitrile, with 0.1% of acetic acid and a flow rate of 1 mL/min. The eluent flow was splitted to the mass spectrometer. Mass spectrometry was carried out using a Bruker Esquire~LC instrument (Bruker Daltonik, Bremen, Germany), with electrospray ionization (ESI) operating in the positive ion mode. Following parameters were used: drying gas nitrogen 8 L/min, nebulizer 35 psi, dry gas heating 350 °C, HV capillary 4000 V, HV EndPlate offset −500 V. For full experimental details of the synthesis and characterization of described compounds see Chemical Synthesis and Characterization.

### 4.3. Chemical Synthesis and Characterization

#### 4.3.1. 3-(1*H*-Indol-3-yl)-4-(3,4,5-trimethoxyphenyl)-1*H*-pyrrole-2,5-dione (**1**)

**Figure 9:**
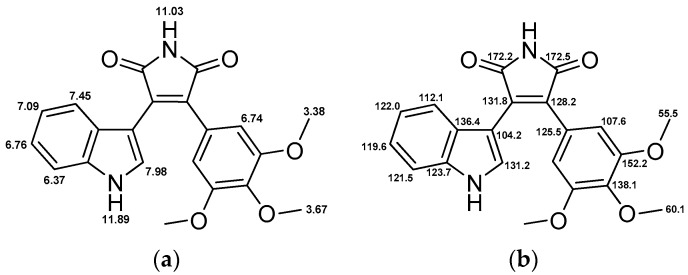
Chemical structure of **1** and relevant NMR shifts for (**a**) ^1^H-NMR; and (**b**) ^13^C-NMR.

2-(3,4,5-Trimethoxyphenyl)acetamide (10 mmol, 2.25 g) was dissolved in dry THF (30 mL) under nitrogen atmosphere and the reaction mixture was cooled to 0 °C. Ethyl 2-(1*H*-indol-3-yl)- 2-oxoacetate (13 mmol, 2.82 g) dissolved in dry THF (40 mL) was added dropwise. Afterwards, potassium *tert*-butoxide solution (1m in THF, 40 mmol, 40 mL) was added. Subsequently, the deep purple reaction mixture was stirred for 6 h at room temperature. Quenching of the reaction with saturated ammonium chloride solution (40 mL) changed the color to orange. After addition of ethyl acetate (50 mL), the solution was stirred for another 15 min. After filtration, the organic layer was washed with brine, dried over Na_2_SO_4_ and evacuated. The crude product was purified by flash silica gel chromatography with a gradient of petroleum ether and ethyl acetate to give an orange solid (5.9 mmol, 2.25 g, 59%). C_21_H_18_N_2_O_2_ (M*_r_* 378.38, Figure 9). Purity (HPLC) > 98%; m.p. 243 °C; ^1^H-NMR (DMSO-*d*_6_): δ 3.38 (s, 6H), 3.67 (s, 3H), 6.37 (d, ^3^*J* = 8.0 Hz, 1H), 6.74 (s, 2H), 6.76 (t, ^3^*J* = 7.9 Hz, 1H), 7.09 (t, ^3^*J* = 7.6 Hz, 1H), 7.45 (d, ^3^*J* = 8.0 Hz, 1H), 7.98 (d, ^3^*J* = 1.7 Hz, 1H), 11.03 (s, 1H), 11.89 (bs, 1H); ^13^C-NMR (DMSO-*d*_6_): δ 55.5, 60.1, 104.2, 107.6, 112.1, 119.6, 121.5, 122.0, 123.7, 125.5, 128.2, 131.2, 131.8, 136.4, 138.1, 152.2, 172.2, 172.5; LC-MS (ESI): *m*/*z* 379 [MH]^+^.

#### 4.3.2. 5,6,7-Trimethoxybenzo[*a*]pyrrolo[3,4-*c*]carbazole-1,3(2*H*,8*H*)-dione (**3**)

**Figure 10:**
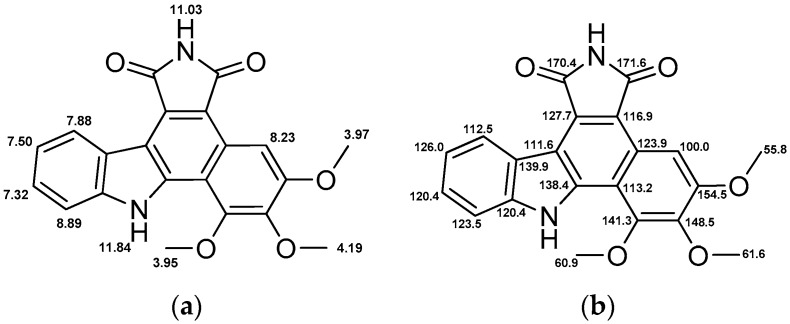
Chemical structure of **3** and relevant NMR shifts for (**a**) ^1^H-NMR; and (**b**) ^13^C-NMR.

3-(1*H*-Indol-3-yl)-4-(3,4,5-trimethoxyphenyl)-1*H*-pyrrole-2,5-dione (**1**, 0.3 mmol, 114 mg) was dissolved in DMSO (20 mL) and irradiated with an LED reactor at 365 nm (5.4 W) for 30 min. Ethyl acetate (100 mL) was added and washed thoroughly with water, dried over Na_2_SO_4_ and concentrated. Purification by flash silica gel chromatography with a gradient of petroleum ether and ethyl acetate afforded an orange solid (0.03 mmol, 12 mg, 11%). C_21_H_16_N_2_O_5_ (M*_r_* 376.36, Figure 10). Purity (HPLC) >98%; m.p. 275 °C; ^1^H-NMR (DMSO-*d*_6_): δ 3.95 (s, 3H), 3.97 (s, 3H), 4.19 (s, 3H), 7.32 (t, ^3^*J* = 7.6, 1H), 7.50 (t, ^3^*J* = 7.6, 1H), 7.88 (d, ^3^*J* = 8.1, 1H), 8.23 (s, 1H), 8.89 (d, ^3^*J* = 7.8, 1H), 11.03 (s, 1H), 11.84 (s, 1H); ^13^C-NMR (DMSO-*d*_6_): δ 55.8, 60.9, 61.6, 100.0, 111.6, 112.5, 113.2, 116.9, 120.4, 120.4, 123.5, 123.9, 126.0, 127.7, 138.4, 139.9, 141.3, 148.5, 154.5, 170.4, 171.6; LC-MS (ESI): *m*/*z* 377 [MH]^+^.

#### 4.3.3. 1-(4,5-Dimethoxy-2-nitrobenzyl)-3-(1*H*-indol-3-yl)-4-(3,4,5-trimethoxyphenyl)-1*H*-pyrrole-2,5-dione (**4**)

**Figure 11:**
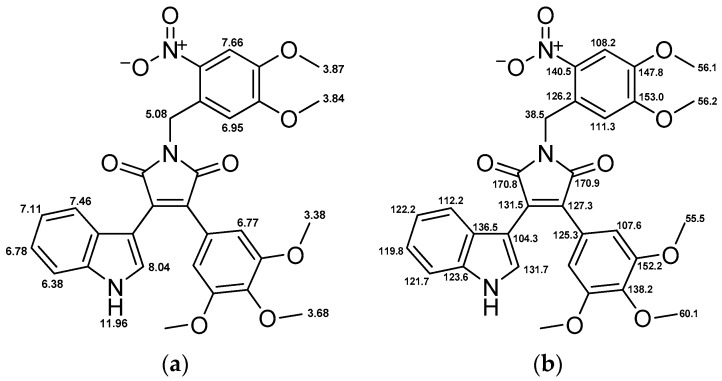
Chemical structure of **4** and relevant NMR shifts for (**a**) ^1^H-NMR; and (**b**) ^13^C-NMR.

3-(1*H*-Indol-3-yl)-4-(3,4,5-trimethoxyphenyl)-1*H*-pyrrole-2,5-dione (**1**, 0.5 mmol, 189 mg) and K_2_CO_3_ (1 mmol, 138 mg) were dissolved in dry DMF (15 mL). 4,5-dimethoxy-2-nitrobenzyl bromide (0.5 mmol, 138 mg) was dissolved in dry DMF (2 mL) and added dropwise to the reaction mixture. After stirring at room temperature for 2 h, the solvent was evaporated and the crude product was redissolved in ethyl acetate, washed with brine, dried over Na_2_SO_4_ and concentrated. Purification by flash silica gel chromatography with a gradient of petroleum ether and ethyl acetate afforded an orange solid (0.27 mmol, 155 mg, 54%). C_30_H_27_N_3_O_9_ (M*_r_* 573.55, Figure 11). Purity (HPLC) >98%; m.p. 205 °C; ^1^H-NMR (DMSO-*d*_6_): δ 3.38 (s, 6H), 3.68 (s, 3H), 3.84 (s, 3H), 3.87 (s, 3H), 5.08 (s, 2H), 6.38 (d, ^3^*J* = 8.1 Hz, 1H), 6.77 (s, 2H), 6.78 (t, ^3^*J* = 7.3 Hz, 1H), 6.95 (s, 1H), 7.11 (t, ^3^*J* = 7.5 Hz, 1H), 7.46 (d, ^3^*J* = 8.0 Hz, 1H), 7.66 (s, 1H), 8.04 (d, ^3^*J* = 2.6 Hz, 1H), 11.96 (s, 1H); ^13^C-NMR (DMSO-*d*_6_): δ 38.5, 55.5, 56.1, 56.2, 60.1, 104.3, 107.6, 108.2, 111.3, 112.2, 119.8, 121.7, 122.2, 123.6, 125.3, 126.2, 127.3, 131.5, 131.7, 136.5, 138.2, 140.5, 147.8, 152.2, 153.0, 170.8, 170.9; LC-MS (ESI): *m*/*z* 574 [MH]^+^.

#### 4.3.4. 2-(4,5-Dimethoxy-2-nitrobenzyl)-5,6,7-trimethoxybenzo[*a*]pyrrolo[3,4-*c*]carbazole-1,3(2*H*,8*H*)- dione (**5**)

**Figure 12:**
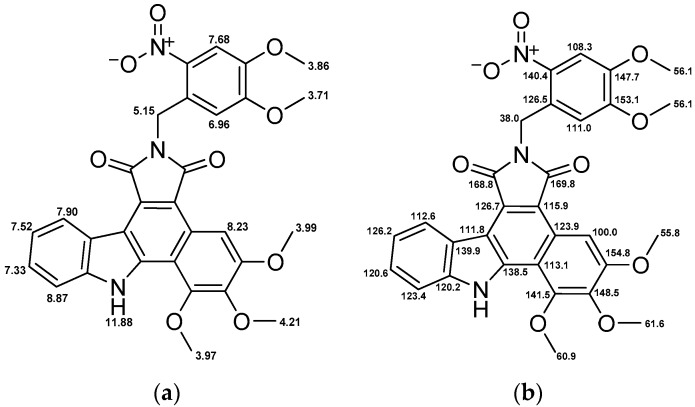
Chemical structure of **5** and relevant NMR shifts for (**a**) ^1^H-NMR; and (**b**) ^13^C-NMR.

5,6,7-Trimethoxybenzo[a]pyrrolo[3,4-*c*]carbazole-1,3(2*H*,8*H*)-dione (**3**, 0.13 mmol, 28 mg) and K_2_CO_3_ (0.38 mmol, 52 mg) were dissolved in dry DMF (5 mL). 4,5-Dimethoxy-2-nitrobenzyl bromide (0.13 mmol, 33 mg) was dissolved in dry DMF (2 mL) and added dropwise to the reaction mixture. After stirring at room temperature for 2 h, the solvent was evaporated and the crude product redissolved in ethyl acetate, washed with brine, dried over Na_2_SO_4_ and concentrated. Recrystallization from ethyl acetate gave an orange solid (0.03 mmol, 19 mg, 26%). C_30_H_25_N_3_O_9_ (M*_r_* 571.53, Figure 12). Purity (HPLC) >98%; m.p. 283 °C; ^1^H-NMR (DMSO-*d*_6_): δ 3.71 (s, 3H), 3.86 (s, 3H), 3.97 (s, 3H), 3.99 (s, 3H), 4.21 (s, 3H), 5.15 (s, 2H), 6.96 (s, 1H), 7.33 (t, ^3^*J* = 7.4 Hz, 1H), 7.52 (t, ^3^*J* = 7.4 Hz, 1H), 7.68 (s, 1H), 7.90 (d, ^3^*J* = 7.6 Hz, 1H), 8.23 (s, 1H), 8.87 (d, ^3^*J* = 7.6 Hz, 1H), 11.88 (s, 1H); ^13^C-NMR (DMSO-*d*_6_): δ 38.0, 55.8, 56.1, 60.9, 61.6, 100.0, 108.3, 111.0, 111.8, 112.6, 113.1, 115.9, 120.2, 120.6, 123.4, 123.9, 126.2, 126.5, 126.7, 138.5, 139.9, 140.4, 141.5, 147.7, 148.5, 153.1, 154.8, 168.8, 169.8; LC-MS (ESI): *m*/*z* 571 [MH]^+^.

#### 4.3.5. 2-(3,4,5-Trimethoxyphenyl)acetamide (**7**)

**Figure 13:**
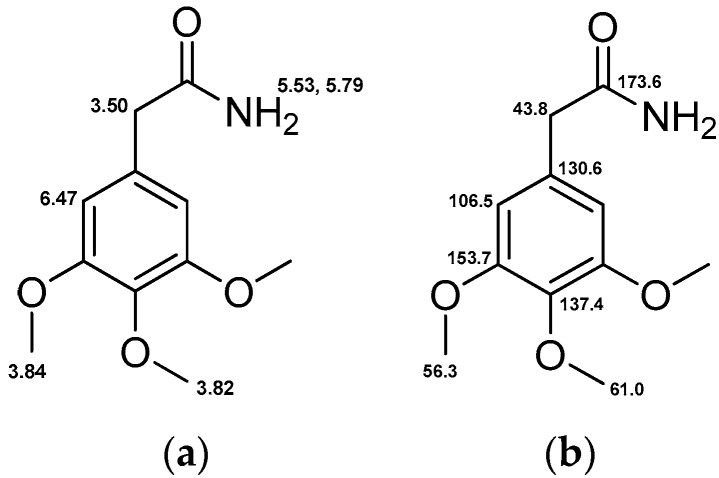
Chemical structure of **7** and relevant NMR shifts for (**a**) ^1^H-NMR; and (**b**) ^13^C-NMR.

2-(3,4,5-Trimethoxyphenyl)acetic acid (20 mmol, 4.6 g) was dissolved in anhydrous THF (30 mL). After addition of thionyl chloride (40 mmol, 3 mL) and a catalytic amount of DMF, the reaction mixture was heated to 40 °C until gas formation was completed (30 min). Subsequently, the solvent and excessive thionyl chloride were removed under reduced pressure. This step was repeated after addition of THF (10 mL). The remaining brown oil was dissolved in DCM (50 mL) and cooled to 0 °C. Next, ammonia solution (25%, 10 mL) was added to the mixture and stirred for 1 h at room temperature. After addition of hydrochloric acid (1m, 20 mL), the organic layer was washed with brine and dried over Na_2_SO_4_. Evacuation and recrystallization from ethanol afforded grey needles (16.8 mmol, 3.86 g, 84%). C_11_H_15_NO_4_ (M*_r_* 225.24, Figure 13). Purity (HPLC) >98%; m.p. 124 °C; ^1^H-NMR (CDCl_3_): δ 3.50 (s, 2H), 3.82 (s, 3H), 3.84 (s, 6H), 5.53 (bs, 1H), 5.79 (bs, 1H), 6.47 (s, 2H); ^13^C-NMR (CDCl_3_): δ 43.8, 56.3, 61.0, 106.5, 130.6, 137.4, 153.7, 173.6; LC-MS (ESI): *m*/*z* 226 [MH]^+^.

#### 4.3.6. Ethyl 2-(1*H*-indol-3-yl)-2-oxoacetate (**10**)

**Figure 14:**
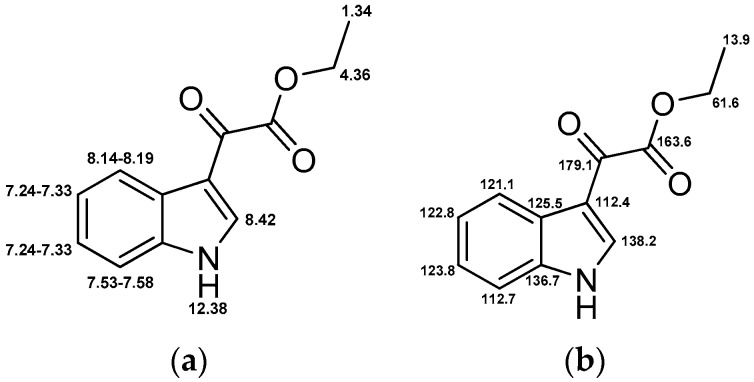
Chemical structure of **10** and relevant NMR shifts for (**a**) ^1^H-NMR; and (**b**) ^13^C-NMR.

Indole (40 mmol, 4.8 g) was dissolved in dry DCM (70 mL) under nitrogen atmosphere and stirred at 0 °C. After dropwise addition of diethylaluminium chloride solution (1m in hexane, 60 mmol, 60 mL), the reaction mixture was stirred for 30 min at 0 °C. Subsequently, ethyl oxalyl chloride (60 mmol, 6.8 mL) was added dropwise followed by stirring for further 3 h. In the next step, ice was carefully added to the reaction mixture for hydrolysis. The organic layer was washed with saturated ammonium chloride solution and brine, dried over Na_2_SO_4_ and concentrated. Purification by flash silica gel chromatography with a gradient of petroleum ether and ethyl acetate afforded light-pink needles (21.9 mmol, 4.75 g, 55%). C_12_H_11_NO_3_ (M*_r_* 217.22, Figure 14). Purity (HPLC) >98%; m.p. 186 °C; ^1^H-NMR (CDCl_3_): δ 1.34 (t, ^3^*J* = 7.1 Hz, 3H), 4.36 (q, ^3^*J* = 7.1, 2H), 7.24–7.33 (m, 2H), 7.53–7.58 (m, 1H), 8.14–8.19 (m, 1H), 8.42 (d, ^3^*J* = 3.3, 1H), 12.38 (bs, 1H); ^13^C-NMR (CDCl_3_): δ 13.9, 61.6, 112.4, 112.7, 121.1, 122.8, 123.8, 125.5, 136.7, 138.2, 163.6, 179.1; LC-MS (ESI): *m*/*z* 218 [MH]^+^.

### 4.4. Photochemical Characterization

#### 4.4.1. UV/Vis Absorption Spectra

Spectra were recorded on UV/Vis spectrophotometer Varian Cary^®^ 50 Scan (Agilent Technologies). UV/Vis absorbance was measured in DMSO. Concentration of compounds was 0.1 mM. Subsequently, graphs were normalized on basis of area under the curve between 415 nm and 520 nm.

#### 4.4.2. Fluorescence Emission Spectra

Spectra were recorded on LS55 Fluorescence spectrometer (Perkin Elmer, Waltham, MA, USA). The excitation and emission slits were set to 5 nm. The fluorescence was measured in DMSO. Concentration of compounds was 0.5 mM. The emission spectrum of carbazole 3 was recorded at constant excitation with 480 nm. Caged carbazole 5 was excited at 500 nm.

#### 4.4.3. UV Stability

Compounds **1** and **3** were dissolved in DMSO (1 mM) and irradiated at 365 nm (LED source: 12× Nichia NCSU033B, Sahlmann Photochemical Solutions, Bad Segeberg, Germany, 100%, 5.4 W) up to 10 min. Aliquots were diluted 1:10 with methanol and analyzed by HPLC. Additional to HPLC analysis LC-MS was used to confirm compound identity.

#### 4.4.4. Photoactivation

Compounds **4** and **5** were dissolved in DMSO (1 mM) and the solutions were irradiated at 365 nm (LED source: 12× Nichia NCSU033B, Sahlmann Photochemical Solutions, 100%, 5.4 W) up to 10 min. After 0.25, 0.5, 0.75, 1, 2, 3, 5 and 10 min of irradiation samples were taken. Aliquots were diluted 1:5 with methanol and subsequently analyzed by HPLC. Additional to retention time LC-MS was used to proof identity.

### 4.5. Kinase Assays

#### 4.5.1. Determination of IC_50_ Values

The VEGFR-2 IC_50_ profile for **1**, **3**, **4** and **5** was determined using VEGFR-2 protein kinase by a radiometric ^33^PanQinase^®^ assay [40]. IC_50_ values were measured by testing 10 semi-log concentrations of each compound in the range from 1 × 10^−4^ M to 3 × 10^−9^ M, for single samples. Prior to testing, the compounds were dissolved to prepare 1 × 10^−2^ M stock solutions in 100% DMSO. The final DMSO concentration in the reaction cocktails was 1% in all cases. Analyses were performed by ProQinase (Freiburg, Germany).

#### 4.5.2. Kinase Profiling

Compounds **1** and **3** were screened against 79 kinases. The used method was a radioactive filter binding assay using ^33^P ATP, for details see references [41,42]. The substances were dissolved in DMSO at tested concentration of 0.1 µM. The mean percentage residual kinase activity and standard deviations of assay duplicates were determined. Analyses were performed by the International Center for Kinase Profiling at the University of Dundee, UK.

### 4.6. CellularAssays

#### 4.6.1. Cell Culture

PC-3 cells were purchased from CLS Cell Lines Service GmbH (Eppelheim, Germany). The cells were grown in DMEM:Hams F12 (1:1) medium with 5% FCS. PC-3 cells were incubated in a 5% CO_2_ humidified atmosphere at 37 °C.

#### 4.6.2. Proliferation Assays

The cells were grown in cell flasks until approximately 90% confluence and then seeded to give 13000 cells in 100 μL per well into 96-well CulturePlates™ (Perkin Elmer). In addition to the test plates, one plate was prepared for reference measurement at day zero. All plates were incubated for 24 h at 37 °C in a humidified atmosphere with 5% CO_2_. Compounds **1**, **3**, **4**, and **5** were dissolved in 100% DMSO (*v*/*v*) and added to the test plates. The final DMSO concentration in the assay was 0.5% (*v*/*v*). Viability of the cells in the day zero control plates were determined on the same day without adding any compounds. For viability measurement the resazurin assay was used. The shift in the fluorescence signal was measured at the LS55 Fluorescence spectrometer (Perkin Elmer). For the photoactivation experiments the test plates were irradiated at 365 nm for 5 min (LED source: 8× Nichia NCSU033B, Sahlmann Photochemical Solutions, 50%, 1.1 kW/m^2^). Test plates were incubated for further 48 h and cell viability was defined as described above. Measured raw data was converted into percent of cell growth by using the high control (0.5% DMSO (*v*/*v*) without compound) and the day zero control. For dose-response studies, 11 different concentrations of compounds were tested in duplicates. IC_50_ values were calculated using the 4-parameter logarithmic fit option of GraphPad Prism 5.

#### 4.6.3. Cell Staining

PC-3 cells were stained with the carbazole **3** and its caged derivative **5**. For this purpose, the cells were seeded to give 15,000 cells in 50 µL per well into 96-well half area microplate (Ref.: 675986, Greiner bio-one, Kremsmünster, Austria). The plate was incubated for 48 h at 37 °C in a humidified atmosphere with 5% CO_2_. Compounds **3** and **5** were dissolved in DMEM:Hams F12 (1:1) medium with 5% FCS and added to the cells to give the endconcentration of 10 µM, respectively. The cells were incubated with the compounds for 30 min at 37 °C. After that, the cells were washed twice with PBS and fixed with 3.3% formalin for 15 min. Then the cells were washed twice with PBS again and the cell nuclei were counterstained with 1 µg/mL DAPI in PBS. After 30 min incubation at 37 °C, the cells were washed with PBS and the fluorescence images were taken at the ImageXpress^®^ Micro XL (Molecular Devices, Sunnyvale CA, USA). The magnification was 60×. Following filter sets were used for the visualisation of compounds (Table 3).

## Figures and Tables

**Table 1:** Inhibitory activity of parent and caged compounds in a VEGFR-2 kinase assay (ProQinase, Freiburg, Germany) [40]. The IC_50_ values are presented.

Compound	VEGFR-2 IC_50_ (µM)
**1**	0.005
**3**	0.152
**4**	4.590
**5**	44.80

**Table 2:** Antiproliferative activity of tested compounds in cellular growth assays with PC-3 cells, without and after irradiation at 365 nm (1.1 kW/m^2^). The GI_50_ values (50% cellular growth inhibition) are presented. “Not reached”: The tested compound did not inhibit the cell growth to 50%.

Compound	GI_50_ without Irradiation (µM)	GI_50_ after Irradiation (µM)
**1**	6.4	2.9
**3**	0.2	0.4
**4**	not reached	0.1
**5**	34.6	0.2

**Table 3:** Filter sets used for the cell staining.

Compound	Filter Set	Exciter (nm) (Center/Bandwidth)	Emitter (nm) (Center/Bandwidth)
DAPI	DAPI	377/50	447/60
3	GFP	472/30	520/35
5	YFP	500/24	542/27

## Supplementary Materials

Supplementary materials can be accessed at: http://www.mdpi.com/1420-3049/21/5/570/s1.

## Abbreviations

DAPI: 4′,6-diamidino-2-phenylindole
DMNB: 4,5-dimethoxy-2-nitrobenzyl
e.g.: for example
HPLC: high performance liquid chromatography
LC: liquid chromatography
MS: mass spectrometry
PBS: phosphate buffered saline
PPG: photoremovable protecting group
resp.: respectively
UV: ultraviolet
VEGF: vascular endothelial growth factor
VEGFR: vascular endothelial growth factor receptor
