# Supplementary material for: Photoactivatable Caged Prodrugs of VEGFR-2 Kinase Inhibitors
Source: Molecules. 2016 Apr 29;21(5):570. doi: 10.3390/molecules21050570 (PMC6274539; doi:10.3390/molecules21050570)
Supplement: Supplementary file 1 [file molecules-21-00570-s001.pdf]

Supplementary Materials: Photoactivatable Caged Prodrugs of VEGFR-2 Kinase Inhibitors

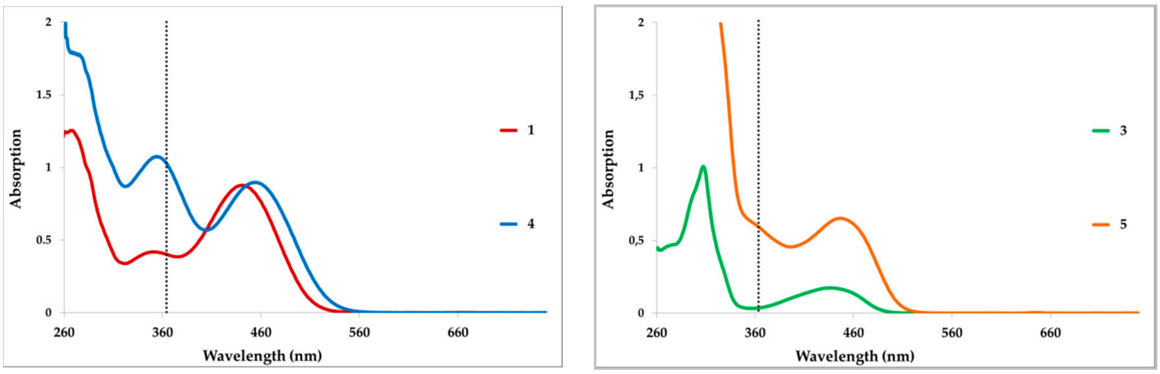

(**a**) (**b**)

Figure S1. Raw UV/Vis absorption spectra of 0.1 mM compounds in DMSO. (**a**) UV/Vis absorption spectra of maleimide (**1**) (red line) and its caged prodrug **4** (blue line). (**b**) UV/Vis absorption spectra of carbazole (**3**) (green line) and its caged analogue **5** (orange line). The black dotted line in both diagrams flags 365 nm as the wavelength used for irradiation of caged compounds.

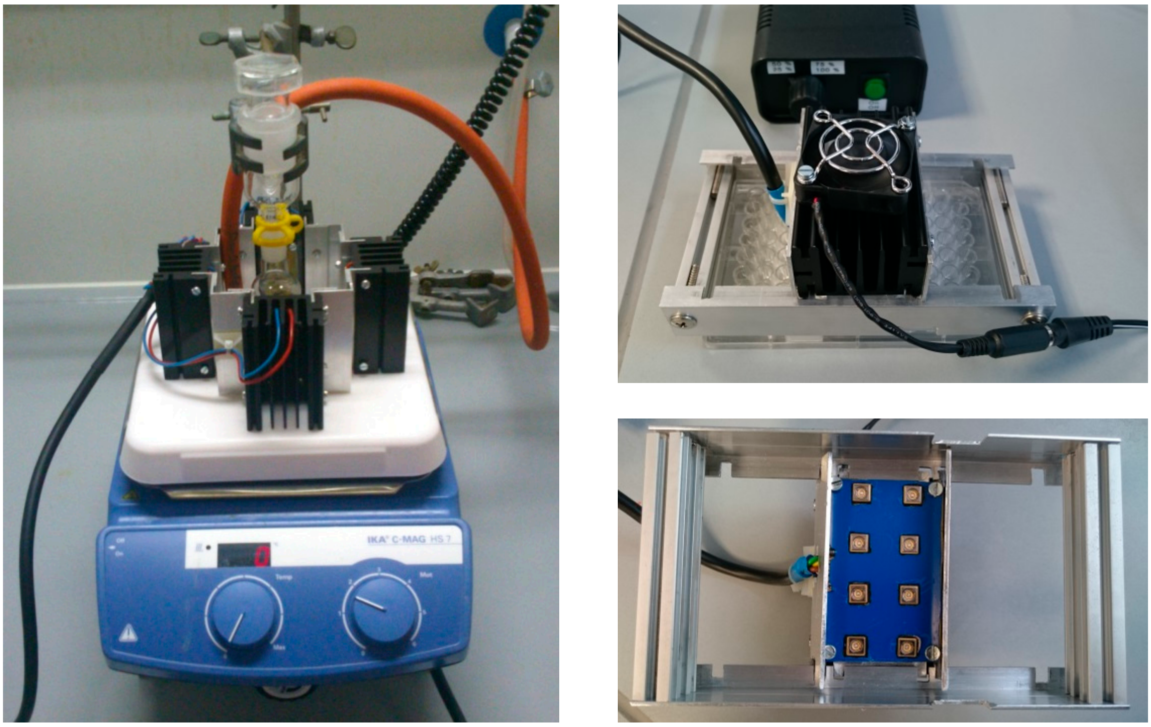

(**a**) (**b**)

Figure S2. UV sources used for irradiation at 365 nm. (**a**) LED reactor used for irradiation of solutions in round bottom flasks. (**b**) LED device for the irradiation of 96-well microtiter plates. Eight LEDs (365 nm) irradiate four rows of wells (32 wells) at once. Accordingly three irradiation cycles are needed for the entire plate. See Figure S3 for Technical setup and emission characteristics.

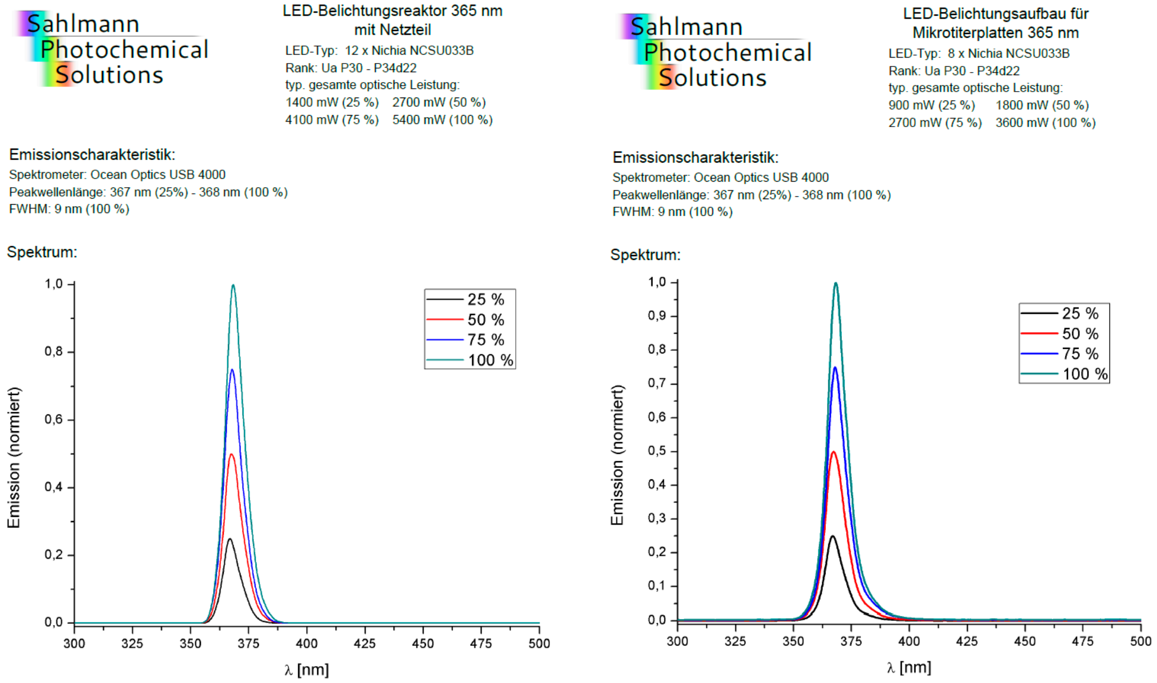

(**a**) (**b**)

Figure S3. Emission characteristics of the used UV sources. (**a**) LED reactor used for irradiation of solutions in round bottom flasks. The reactor consists of 12 LEDs with an emission maximum at a wavelength of 365 nm. The intensity can be adjusted to four levels (25, 50, 75 and 100%). The corresponding optical power in mW is shown. (**b**) LED device for irradiation of 96-well microtiter plates. There are also four intensity levels available.

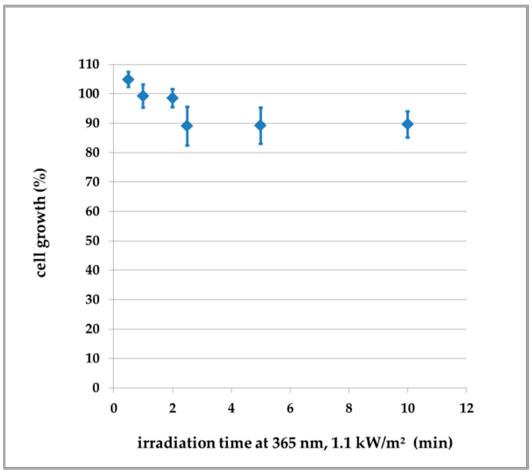

Figure S4. Cell growth of PC-3 cells depending on the irradiation time. Cells were irradiated at 365 nm (1.1 kW/m^2) for indicated periods of time. Cell growth was determined after 48 h.

(**a**) (**b**)

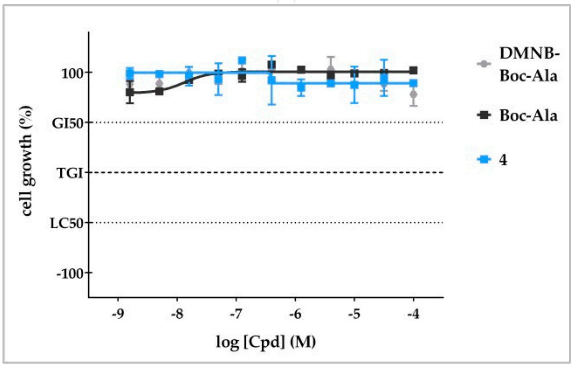

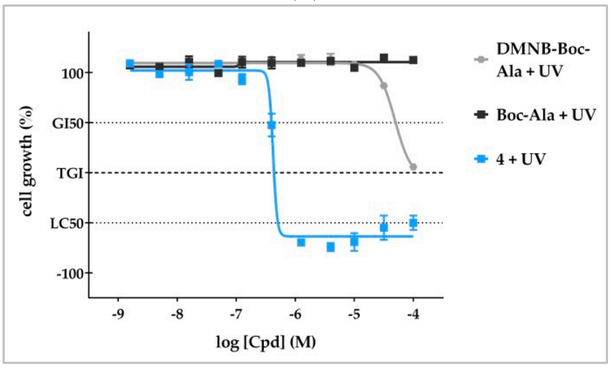

(**c**) (**d**)

Figure S5. Effect of the photo released PPG on the cell proliferation of PC-3 cells. (**a**) Chemical structure of the BOC-protected L-alanine = Boc-Ala. (**b**) Chemical structure of the DMNB-protected Boc-Ala = DMNB-Boc-Ala. (**c**) Dose-response-curves of the Boc-Ala, DMNB-Boc-Ala and caged compound 4 in cell viability assay using the PC-3 cells without UV irradiation. Cell growth was determined after 48 h incubation with the compounds. The tested compounds do not show any anti-proliferative effects. (**d**) Dose-response curves of 4, Boc-Ala and DMNB-Boc-Ala in the same assay as in (c) with UV irradiation. Cells were incubated with compounds for 1 h and then irradiated at 365 nm (1.1 kW/m^2) for 5 min. Cell growth was determined after 48 h. Herein, the DMNB-Boc-Ala exhibits anti-proliferative efficacy in concentrations above 30 μM providing evidence for the cytotoxicity of the cleaved PPG at higher concentrations. GI_{50} = 50% growth inhibition; TGI = total growth inhibition; LC_{50} = 50% lethal concentration; the dose response curves were fitted using the 4-parameter logistic fit option of GraphPad Prism 5; n = 2 and error bars represent standard deviation.

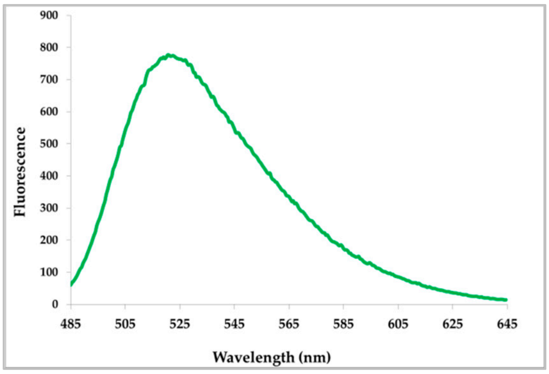

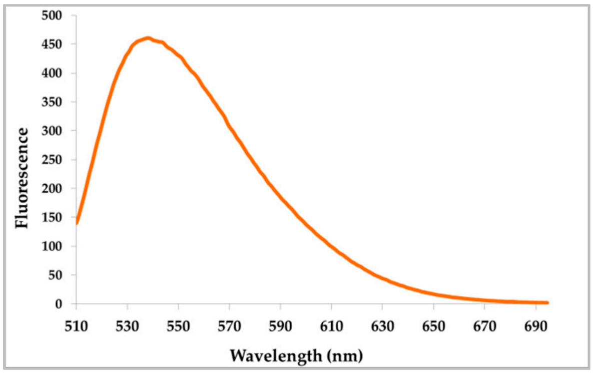

(**a**) (**b**)

Figure S6. Raw fluorescence emission spectra of 0.5 mM compounds **3** and **5** in DMSO. (**a**) Fluorescence emission spectrum of carbazole (**3**); Excitation wavelength: 480 nm. (**b**) Fluorescence emission spectrum of caged carbazole (**5**); Excitation wavelength: 500 nm.

Table S1. Kinase profiling of maleimide **1** and carbazole **2**. The inhibitory effect of active compounds was tested on a panel of 79 kinases. The residual activity of kinases was measured after incubation with 0.1 μM of each compound. The data is portrayed as mean percentage activity and standard deviation of assay duplicates. (MRC, Dundee, Scotland).

Kinase	Compound 3	Deviation	Compound 1	Deviation
MKK1	67	5	54	27
ERK1	91	25	100	5
ERK2	93	0	94	5
JNK1	96	5	96	13
JNK2	93	16	78	14
p38a MAPK	78	12	89	0
P38b MAPK	89	17	86	8
p38g MAPK	86	7	91	2
p38s MAPK	90	17	108	0
ERK8	11	1	46	5
RSK1	51	6	96	7
RSK2	62	6	91	1
PDK1	39	1	68	4
PKBa	81	16	66	4
PKBb	91	9	98	2
SGK1	52	15	75	5
S6K1	28	1	65	4
PKA	90	5	93	13
ROCK 2	88	6	97	2
PRK2	52	2	84	4
PKCa	90	14	113	5
PKC zeta	98	2	102	6
PKD1	60	9	100	12
MSK1	55	10	69	1
MNK1	79	4	80	0
MNK2	81	1	90	15
MAPKAP-K2	95	4	94	2
PRAK	85	2	91	7
CAMKKb	49	3	81	3
CAMK1	94	18	107	1
SmMLCK	30	5	60	17
PHK	41	6	73	8
CHK1	34	3	72	0
CHK2	63	2	64	9
GSK3b	5	1	21	2
CDK2-Cyclin A	28	4	59	5
PLK1	49	5	79	9
PLK1 (Okadaic Acid)	83	0	85	2
Aurora B	47	14	84	12
AMPK	75	13	78	8
MARK3	73	3	90	5
BRSK2	66	2	94	8
MELK	55	7	74	13
CK1	78	2	86	5
CK2	91	10	94	9
DYRK1A	26	0	73	2
DYRK2	17	0	57	15

DYRK3	46	5	85	6
NEK2a	91	6	87	1
NEK6	104	9	96	2
IKKb	113	25	102	7
PIM1	5	1	14	1
PIM2	115	12	115	5
PIM3	4	2	5	1
SRPK1	83	5	85	1
MST2	24	2	53	0
EFK2	109	15	104	3
HIPK2	7	1	23	2
PAK4	55	6	75	3
PAK5	84	4	105	1
PAK6	99	2	97	5
Src	86	1	81	0
Lck	91	4	91	2
CSK	99	1	138	8
FGF-R1	92	1	69	9
IRR	41	0	58	1
EPH A2	109	4	104	6
MST4	69	3	94	7
SYK	86	16	85	5
YES1	88	5	84	14
IKKe	63	8	80	3
TBK1	85	7	87	1
IGF1-R	65	4	90	24
VEG-FR	37	2	10	0
BTK	90	1	93	14
IR-HIS	88	15	101	11
EPH-B3	133	4	104	14
TBK1 (DU12569)	73	2	88	5
IKK epsilon (14231)	94	5	85	7
